# Actions of Bisphenol A on Different Feto-Maternal Compartments Contributing to Preterm Birth

**DOI:** 10.3390/ijms23052411

**Published:** 2022-02-22

**Authors:** Manuel S. Vidal, Ramkumar Menon, Gracia Fe B. Yu, Melissa D. Amosco

**Affiliations:** 1 College of Medicine, University of the Philippines Manila, Manila 1000, Philippines; 2 Division of Basic Science and Translational Research, Department of Obstetrics and Gynecology, The University of Texas Medical Branch at Galveston, Galveston, TX 77555, USA; ra2menon@utmb.edu; 3 Department of Biochemistry and Molecular Biology, University of the Philippines Manila, Manila 1000, Philippines; grafebyu@gmail.com; 4 Department of Obstetrics and Gynecology, Philippine General Hospital, University of the Philippines Manila, Manila 1000, Philippines; mdamosco@up.edu.ph

**Keywords:** bisphenol, BPA, preterm birth, endocrine-disrupting compounds

## Abstract

Preterm birth remains to be one of the most prevalent obstetric complications worldwide. Since there are multiple etiological factors associated with this disease process, an integrative literature search in PubMed and Scopus databases on possible mechanism of action and effect of bisphenols on exposure on human or animal placental samples in preterm birth was conducted. From 2332 articles on initial literature search, 63 studies were included for full data extraction. Altogether, several pathways were shown to be possibly affected by bisphenols, leading to dysregulations in structural and endocrine foundation in the placenta, potential induction of senescence and failure of decidualization in the decidua, and possible propagation of inflammation in the fetal membranes. Combined, these actions may eventually counteract bisphenol-induced relaxation of the myometrium and promote contractility alongside fetal membrane weakening. In totality, these individual impairments in gestation-critical processes may lead to failure of maintenance of pregnancy, and thus effecting preterm birth.

## 1. Introduction

Annually, 15 million infants are born prior to 37 weeks of gestation, defined by the World Health Organization (WHO) as preterm birth (PTB). Annual rates range from 5–18% and is the top cause of mortality among children < 5 yr of age [1]. Short-term complications may arise and lead to hospital-acquired infections, intraventricular hemorrhage, and necrotizing enterocolitis [2]. Long-term morbidities such as brain injury and abnormal brain development, cardiovascular, and metabolic abnormalities can be apparent down the line and persist until adolescence [3].

About half of preterm births (PTB) are of spontaneous or idiopathic etiologies, in contrast with preterm rupture of membranes (about a third of PTB cases) or elective preterm deliveries (about a fifth of PTB cases). These pathways are not mutually exclusive of each other, since there are redundancies in downstream effectors for each factor listed; moderate changes brought upon by a single factor may also not be adequate to bring about the definitive outcome of premature delivery [4,5]. Despite multiple different etiologies, the common pathway that allows for the early termination of pregnancy comes down to the generation of synchronous and forceful myometrial contractions and the remodeling of the cervical tract to dilatation and effacement similar to the normal physiological process of labor. Since spontaneous PTBs comprise the majority of these cases, determination of the exact pathophysiology becomes an important endeavor to allow possible interventions that can prevent PTB.

Pollutant exposure is one of the proposed etiologies for spontaneous preterm birth [6,7]. In particular, bisphenols, one of the most prevalent pollutants in the environment, have been associated with a risk for decreased gestational age and preterm birth across multiple studies in different countries [8,9,10,11]. The main congener bisphenol A (BPA) is massively produced annually, with more than 6 billion pounds of yield every year used in food and beverage containers as well as epoxy resins for commercial products [12,13,14]. An alternative congener, bisphenol S (BPS), has also been synthesized and is currently used in containers, paper items, and hygiene products [15,16,17,18]. A recent meta-analysis summarizing papers from major databases published since inception to 2020 regarding BPA exposure and preterm birth highlighted that higher BPA exposure (median or geometric mean concentration > 2.16 ng/mL) is linked to almost a two-fold risk of preterm birth as well as decreased gestational period. The association is significant especially for the third trimester elevated bisphenol levels, suggesting that this may be the critical window as to which BPA exerts its effects [19]. However, studies have been limited regarding their specific mechanisms of action in the feto-maternal unit.

In this light, this review explores the potential role of bisphenols in contributing towards a preterm birth phenotype. We will review pieces of evidence from various experimental models regarding observed molecular mechanisms and discuss the possible pathways in which these bisphenols may act to bring about preterm birth. A deeper understanding of the pathophysiology of risk factors associated with premature delivery may aid researchers, physicians, and policymakers in developing primary, secondary, and tertiary preventive measures.

## 2. Results and Discussion

### 2.1. Search Strategy Employed

**Figure 1:**
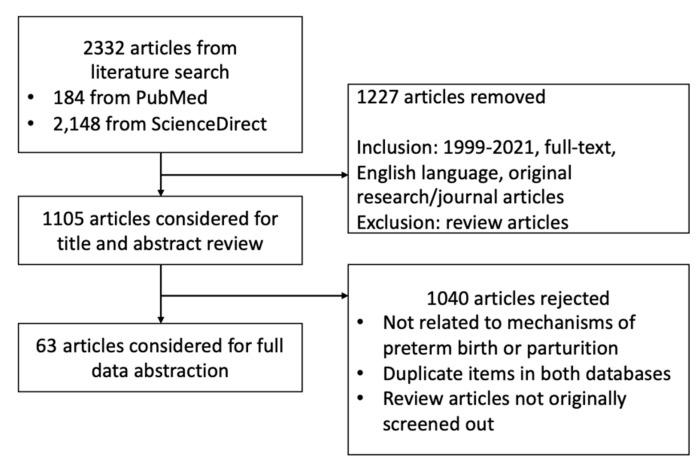
Search strategy employed.

After literature search and applying our inclusion and exclusion criteria, we included a total of 63 original papers for full data abstraction (Figure 1). Outcomes manually collected include type of model used, dose of bisphenols used, and effects observed with significance (*p* < 0.05) (Table 1, Table 2 and Table 3). Due to intermodel congruence of effects, we have designated strong evidences for the following: proliferation, trophoblast motility, trophoblast and placenta morphology, and CRH production in the placenta; inflammation and ER expression in the decidua; and uterine weight, thickness, and contractility in the myometrium. The rest of the effects have been designated as either limited or inconclusive evidence. The data are synthesized in the following subsections of the discussion that follows.

### 2.2. Effects of Bisphenols on the Placenta

BPA effects on various intrauterine tissues are examined primarily as an endocrine disruptor, although many other undetermined effects on “cell fate” may determine underlying pathways and biomarkers of its impact on determining pregnancy outcome. The placental cells express estrogen receptors (ERs), mainly ER⍺ and Erβ [20]. This is important since estrogen is a particular signal for cellular growth and development. Reports vary in the effects of lower concentrations of bisphenols on cellular proliferation possibly initiated by the ER⍺ pathway; increased proliferation has been observed from 10^−5^ up to 10 μM [21,22,23]. Alternatively, BPA-induced estrogen function may be performed via non-classical receptors; one example is GPR30, a transmembrane receptor that is hypothesized to be critical for placentation [21]. In some cell models, such as HTR-8/Svneo cells and primary cytotrophoblast cells, there appears to be no effect at all [24,25]. Higher BPA doses have consistently demonstrated an observable decrease in proliferation and apparent cytotoxicity beyond 10–100 μM [26,27,28,29]. These cytotoxic effects may be mediated by the estrogen-related receptorɣ1 (ERRɣ1), since abolishing the expression of ERRɣ1 via silencing RNA (siRNA) rescues trophoblast cells from reduced proliferation [22,30]. MicroRNAs (miRNAs) may also play nonclassical roles in the suppression of cell proliferation; miR-146a expression is induced by BPA in HTR-8 cells as well as in placentas of pregnant women, with stable overexpression leading to a significant decrease in proliferation [31,32]. Overall, it can be surmised that placental cellular proliferation is consistently suppressed at higher concentrations; effects on lower concentrations may vary depending on the cell type used and experimental setup [21,22,23,24,25,26,27,28].

Low concentrations of BPA (50 nM) promote cell fusion [33]. However, high concentrations of BPA promote dysregulation of the normal placental phenotype. Long-term BPA exposure of rhesus monkey trophoblast stem cells reduced trophoblast invasion, even at non-toxic concentrations [26]. In mouse placenta, BPA causes a decrease in the labyrinthine and spongiotrophoblast zone proportions with narrowing of the intervillous spaces [34,35,36,37]. The exact mechanisms as to how this occurs seems to be debatable; matrix metalloproteinase-2/9 (MMP2/MMP9) seems to be involved, but upregulation or downregulation occurs depending on the model used [34,35,38,39].

BPA has also been reported to reduce blood flow throughout the uteroplacental unit through morphological vessel disruption in animal models, such as impairments in spiral remodeling and branching, and lumen narrowing that may lead to hypoperfusion [40,41]. BPA inhibits basal and vascular endothelial growth factors (VEGF) by downregulating mRNA expression of VEGF and lowering CpG methylation of gene promoters associated with oxidative stress in HTR8/SVneo cells [42,43]. Decreased migration and attachment have also been observed across a wide range of concentrations, which limit the optimal invasion necessary for sufficient placental formation [24,26,35,38]. Overall, the resulting gradual uteroplacental insufficiency from suboptimal placentation may provide inadequate oxygenation that can trigger eventual feto-maternal stress.

Interestingly, BPA decreases the levels of estrogen and progesterone production in cultured placental cells [28]. In JEG-3 cells, BPA has been shown to upregulate the protein expression of the *CYP1A1* gene, important for detoxification of environmental toxins, and downregulate the protein expression of the *CYP19A1* gene, a player in the in situ conversion of androgens to estradiol in the placenta [44,45]. Similarly, *CYP11A1,* which converts cholesterol to pregnenolone, was also demonstrated to be downregulated after BPA exposure. [28] There has also been a documented decrease in aromatase activity even in non-toxic BPA doses, suggesting that direct interaction of BPA with the gene product is possible [46,47]. Decreased hormone production in the placenta is paralleled by a decrease in ER and progesterone receptor (PR) expression and activity in higher doses [48,49,50,51,52]. However, the lack of exploration on in vivo models on steroidogenesis provides us with a limited view of the effects of BPA on steroid receptor expression.

A central player in parturition is the expression of corticotropin-releasing hormone (CRH), classically leading to eventual myometrial contractions and decidual production of prostaglandins as an integral indicator of fetal stress [53,54,55]. In JEG-3 cells, CRH messenger RNA (mRNA) expression has been found to increase at concentrations of BPA > 25 µM, with downstream actions mediated by protein kinase C as seen in mice placenta [55,56]. CRH also modulates placental steroidogenesis by upregulating enzymes involved in estrogen synthesis while decreasing progesterone production, assisting with the quiescence-to-contractility transition [57,58]. However, no mechanistic studies have been proposed yet regarding the relationship between preterm birth, bisphenols, and urocortins.

Inflammatory mediators in the placenta, such as interleukin (IL)-1β and IL-6, have been demonstrated to increase post-exposure to bisphenol in a dose-dependent manner [59]. In sheep placenta, there was a demonstrable increase in oxidative stress markers and IL-1βs during mid-gestation [60]. These direct synthesis of prostaglandins and metalloproteinases, that leads to eventual parturition [61,62,63]. The placenta may respond via increasing antioxidant activity, such as post-exposure increases in glutathione levels; however, long-term inflammation may overcome any potential acute anti-inflammatory response [64]. However, most of the studies involving inflammatory signaling come from cellular studies, and so these remain inconclusive until validated in mice/humans.

Exposure to BPA has also been proven to cause dysregulation in protein cargo of placental exosomes, which are small extracellular vesicles hypothesized to perform signaling and/or effector actions between individual organs [65]. Proteomic analysis of placental exosomes from BPA-exposed explants showed upregulation in proteins biologically functional for organismal death, morbidity or mortality, and necrosis, and concomitant downregulation of proteins for cell viability and survival as well as migration and spread. Among them, p38 MAPK has been discussed extensively, since its packaging into exosomes may be correlated with cellular response for damage [66]. However, this is a relatively new subject and further experiments on human or animal tissues are needed to validate this response.

Overall, BPA has been shown to reduce cellular proliferation and growth in high doses (≥10–100 μM), while affecting trophoblast motility and morphology (1–100 μM) that may lead to reduction of placental blood flow. Other effects have been noted as well, such as reduction of steroidogenesis, impairment of syncytialization, activation of inflammation, and placental exosome changes; however, these variable effects across different models need to be validated in more robust endeavors.

### 2.3. Effects of Bisphenols on the Decidua

Bisphenols have been shown to induce inflammatory changes in the decidua. For instance, chronic BPA exposure reduced *Hand2* expression, a transcription factor critical for decidualization. This may result in a decrease in IL-15 expression, leading to failure of uterine natural killer cells to eliminate senescent decidual cells [67,68]. This accumulation of senescent decidual cells may result in a local pro-inflammatory environment [69]. In human endometrial stromal cells (ESCs), BPA exposure induces expression of tumor necrosis factor alpha (TNF-ɑ), IL-6, and IL-1β [70]. The latter, along with thrombin, downregulates the expression of *Hoxa10* and the associated *Hoxa11* genes in decidual cells, leading to preterm labor [71]. IL-1β has also been shown to significantly amplify the expression of MMPs, resulting in a matrix-degrading cascade targeting the surrounding matrix [72]. IL-6 expression within the decidua promotes local monocyte differentiation into functional macrophages [73]. These inflammatory changes may contribute to overall prostaglandin production that leads to membrane weakening and eventual preterm birth.

Decidualization involves steroid receptor hormones interactions. BPA has been shown to upregulate levels of ERs and PRs upon exposure to low concentrations of BPA (up to 1 μM), but higher doses correlate with decreased expression [74,75,76,77]. *Hoxa10* is also downregulated upon chronic BPA exposure, resulting in impaired steroid responsiveness of the decidual stroma; concomitant upregulation of steroid receptor corepressor SMRT also occurs in endometrial stromal cells in exchange for promotion of trophoblast invasion [78]. A fall in progesterone responsiveness may trigger a pro-inflammatory decidual response, potentially contributing to the overall senescent phenotype. *Hoxa10* reductions are also correlated with increased enhancer of zeste homolog 2 (EZH2) and decreased mixed-lineage leukemia 1 (MLL1), transcription factors responsible for decidualization [79]. EZH2 mediates gene suppression via histone methylation H3K27me3, while MLL1 allows for transcription via histone trimethylation H3K4me3 [80]. As decidual cells are responsive to ER and PR expression changes, the aforementioned effects must take into consideration that levels may vary across multiple models. Overall, evidence points to the existence of a possible dose- and time-dependence on ER and PR expression in decidual cells upon BPA exposure, and these mechanisms should be further explored.

Steroidogenesis, which is naturally important for decidualization, is also affected by BPA exposure. In ESCs, exposure to BPA results in a decrease in P450 side-chain cleavage (P450scc) enzyme expression, which mediates cholesterol to pregnenolone conversion for progesterone and estrogen synthesis [75,81]. Moreover, hydroxysteroid-17β-dehydrogenase 1 and 2 (HSD17β1 and HSD17β2) expression is also downregulated upon BPA exposure, interfering with estrone and estradiol interconversion [75].

Cell cycle arrest may be another factor that affects decidualization status upon bisphenol exposure. At lower concentrations (0.01 nM to 0.01 μM) BPA-exposed ESCs seem to be arrested at the G2/M phase [82]. However, at higher concentrations (~45 μM–90 μM), BPA markedly downregulated the expression of *CCND2*, the gene encoding for cyclin D2, resulting to a significant fraction of cells arrested in the G1 phase [83,84].

All of these impairments in decidualization are reflected in alterations of decidualization markers. In ICR mice, oral administration of BPA in early pregnancy decreases the levels of desmin and serum/glucocorticoid regulated kinase 1 (SGK1) levels, proteins expressed by decidualizing cells [85]. In human endometrial cells, at low concentrations (<0.01 μM), there is an observed increase in prolactin (PRL) and insulin-like growth factor binding protein 1 (IGFBP1) expression in ESCs [86]. However, at 1 μM, there is a significant reduction of mRNA levels of PRL [74]. At higher concentrations (10 μM), the expression levels of both markers do not significantly differ versus control [86]. At even higher concentrations (>50 μM), however, there was an increase in IGFBP1 levels but not that of PRL [75]. There seems to be a bimodal action of BPA on decidual markers, with concentrations at the window of relevance (between 0.01 μM and 10 μM) providing suppression of decidual markers. However, more standardized experiments are warranted with regards to decidualization and bisphenol exposure.

BPA also induces the expression of decidual *Egr1*, a critical gene for decidualization under estrogen control [87,88]. *Egr1* has been documented to be upregulated in maternal plasma of preterm delivery patients [89]. Decreased estrogen receptivity may lead to possible aberrant overexpression of the gene, leading to (1) inhibition of the decidualization process as evidenced by a significant decrease in decidual/trophoblast PRL-related protein (Dtprp), and (2) increase in *Cox-2* expression that may result to excessive prostaglandin and metalloproteinase synthesis [88,90].

BPA effects on the decidua may also contribute to impaired placentation observed in the previous studies. A decrease in *Hoxa10* in the decidua is critical to allow trophoblast invasiveness [91]. As we have concluded in the previous section, improvement of trophoblast invasion by decidual factors does not necessarily result in overall improved placentation. Excessive activity of MMPs post-exposure to bisphenol may lead to reductions of placental layers, as observed in the HTR-8/SVneo cells [35]. Decidual CXCL8 expression is evidently decreased by BPA exposure, leading to a decrease in trophoblast invasion in in vitro setups. Interestingly, this effect is rescued by the administration of an ER antagonist and a GPR30 antagonist, which connotes that these receptors may be involved in decidual-mediated impairment of invasion post-exposure to BPA [92].

Overall, changes in the decidua upon BPA exposure lead to inflammatory effects as well as an increase in ER expression as observed in different models. Other model-variable effects may include induction of decidual senescence, decreased decidual responsiveness to steroids, and activation of inflammation leading to prostaglandin and MMP synthesis; these remain to be established yet in other experiments.

### 2.4. Effects of Bisphenols on the Myometrium

Little is known about the effects of BPA on the myometrium, but it is generally observed that exposure to a wide range of concentrations (50–500 mg/kg/day) in different routes of administration leads to a uterotrophic phenotype (increased myometrial and stromal thickness, and PCNA immunostaining) across multiple animal models [93,94,95,96,97,98,99]. The effects are hypothesized to be mediated both by ER- and non-ER-mediated pathways [100]. The latter involves nonclassical pathways, such as heat shock protein 72 (hsp72), hsp90ɑ, and homologous glucose-regulated protein 94 (grp94) in ovariectomized mice. These chaperone proteins, aside from protein folding functions, have been associated with ER signaling and, indirectly, with uterotrophism [101].

An increase in uterine weight does not necessarily result in increased uterine contractile force. Curiously, bisphenol has been found to decrease the myometrial contractions in various experiments, despite increasing *OXTR and OXT* expression [102,103]. This dose-dependent decrease in amplitude and frequency of contractions has been suggested to operate via (1)a nitric oxide-involving pathway, and (2)a vesicular acetylcholine transporter (VACht)-mediated pathway; however, these mouse- and porcine-related uterine changes remain to be replicated in human cells [104,105,106].

Moreover, the presence of uterotonins may counteract BPA-induced relaxation. In an ex vivo uterine contraction study using rat uterine tissue, the presence of prostaglandin F2ɑ or oxytocin in spite of BPA exposure restores the force of contraction in a dose-dependent manner [103]. Purportedly, GPR30 activation may also be involved through an increase in oxytocin responsiveness and promotion of actin polymerization through hsp27 and MAPK phosphorylation [107,108]. Genes related to smooth muscle contractility and MAPK signaling have been also found to be upregulated in the presence of uterotonins in an ER-independent manner, although the exact molecular switch remains to be discovered [109,110].

Additionally, a BPA-induced decrease in steroidogenesis in the placenta may result in the downregulation of *Hoxa10/11* in the myometrium. *Hoxa10/11,* repressed by high progesterone levels, causes suppression of mRNAs of various mediators of contraction including the genes for Cx43, COX-2, IL-1β, and IL-6 [111,112]. Increases in inflammatory cytokines may also provide a feed-forward downregulation of *Hoxa10*, which may lead to sufficient contractions in localized areas of the uterus, an increase in MMPs, and eventual preterm birth [112].

Overall, BPA has been consistently observed to increase uterine weight and uterine thickness and induce relaxation in myometrial cells across different models. In essence, we can surmise that all other actions by local units (placenta, decidua, and amnion cells) contribute a far greater effect, and may overcome myometrial relaxation; however, the hypothesis remains to be seen in actual experiments.

### 2.5. Effects of Bisphenols on Fetal Membranes

Very little data is known regarding the direct effects of bisphenols on fetal membranes despite knowledge of its transplacental transfer to the fetal compartment due to a lack of experiments [113]. Nonetheless, a few hypotheses can be generated through isolated studies on oxidative stress. In amnion epithelial cells (AECs), oxidative stress increases hsp70 and p38 MAPK packaging into exosomes; since BPA also induces placental oxidative stress and p38 MAPK exosome packaging, it may be hypothesized that BPA behaves similarly in AECs [59,70,114]. Inhibition of p38 MAPK activation results in senescence reversal and rescue from sterile inflammation [115,116]. AEC exosomes have been found to induce expression of *Cox-43* and *Cox-2* in myocytes after exposure to cigarette smoke extract, suggesting that packaged compounds inside AEC exosomes may be transported to other feto-maternal compartments and may trigger labor-associated changes [66].

Direct inflammation propagation from either decidua or placenta post-exposure to bisphenols may affect fetal membranes as well. In rhesus monkeys, for instance, IL-1β stimulates IL-17 production from chorioamniotic T-cells, propagating the inflammatory response in these layers [61]. On the other hand, IL-6, on its own, does not induce any inflammatory and cellular transition changes in AECs [117]. However, IL-6 stimulates the production of PGE2 with a concomitant decrease in PGDH in AECs, increasing prostaglandins within the fetal membranes especially with a continuous influx of IL-6 from other feto-maternal units [118]. Nonetheless, additional studies are warranted in order to validate the hypothesis that bisphenols may cause inflammation in fetal membranes.

### 2.6. Limitations of the Study

Naturally, due to the ubiquitous nature of BPA especially in commercial products, majority of the studies on the effects of bisphenols on preterm birth circle around BPA itself. Some congeners may act similarly; for instance, BPS exposure has been found to lead to impairment of trophoblast morphogenesis via reductions in e-cadherin expression and blockage of EGFR [119,120]. In some instances, however, these may not follow BPA mechanics; for instance, BPS has been demonstrated to be associated with a decrease in CRH, opening the possibility that (1) different bisphenol analogs differentially act on CRH pathways or (2) CRH analogs such as urocortins may be affected by BPA exposure [121,122]. Moreover, in the context of varying models and physiological environments as to which BPA has been tested in, we cannot definitively conclude that these mechanisms are conserved in the feto-maternal tissues in preterm birth. Nonetheless, by aggregating evidences from these various experiments across different models, this paper provides a stepping stone as to which effects may be expected in actual patients. Hopefully, future experiments may delve deeper into the actions of BPA put forward in this paper and develop more robust methods to elucidate the mechanisms in human tissues. We also observed that majority of these experiments used concentrations that are higher than 2.16 ng/mL (9.5 nM), which is correlated with preterm birth as cited in a previous study. [20] Some studies have touched on this concentration, as seen in Table 1; these are the effects that may occur in human tissues, and future experiments should take this concentration into account.

## 3. Conclusions

Although there have been studies published regarding the possible mechanisms of actions of bisphenols that may contribute to preterm birth, there is a relative lack of coherence due to the use of various in vitro, in vivo, and ex vivo models due to human experimentation barriers. Future studies may be directed towards simulating prenatal and antenatal dosing in order to have a better insight towards short-term, and in particular, long-term effects of these compounds. Doses may also be standardized in terms of concentrations (i.e., calculation of actual human exposure levels) and routes of administration (i.e., either orally in animal models, or through a stable influx of steady-state concentrations in in vitro models). As mentioned, the mean concentration that correlates with preterm birth is around >2.16 ng/mL or 9.5 nM; future experiments may rely on similar or higher values to initiate BPA effects for observation [20]. Lastly, since bisphenols have multiple modes of action across different feto-maternal units, future studies may utilize mechanistic animal models that parallel placental, decidual, myometrial, and fetal membrane endpoints that are duly changed in humans post-exposure to bisphenols. By taking into account the time-, concentration-, and cell-dependent mechanisms of action of bisphenols, these recommendations will help form a more robust notion on the exact pathophysiology of bisphenol exposure in relation to preterm birth.

**Figure 2:**
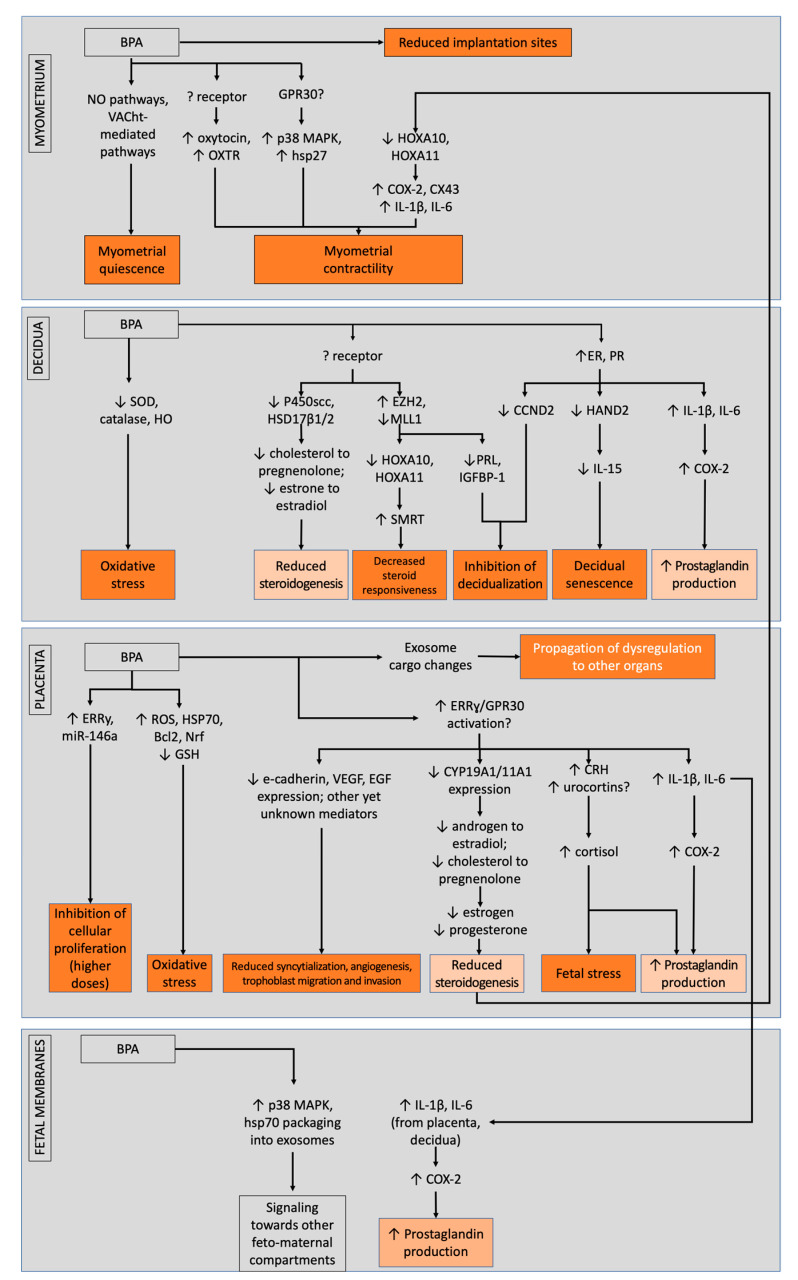
BPA and its mode of actions across different feto-maternal units that possibly contribute to preterm birth, separated between low- and high-dose actions. Light orange boxes denote similar modes of actions between different units, while dark orange boxes denote actions that contribute to the preterm phenotype. Overall, bisphenols cause impairments in the decidua, placenta, and fetal membranes that may counteract myometrial relaxation and promote fetal membrane weakening.

Despite these obstacles, the bits and pieces of information that we gather from these studies should not be discounted. Altogether, several pathways were shown to be possibly affected by bisphenols, leading to dysregulations in structural and endocrine foundation in the placenta, potential induction of senescence and failure of decidualization in the decidua, and possible propagation of inflammation in the fetal membranes; combined, these actions may eventually counteract bisphenol-induced relaxation of the myometrium and promote contractility alongside fetal membrane weakening. In totality, these may lead to failure of maintenance of pregnancy that may lead to preterm birth (Figure 2).

## 4. Materials and Methods

### 4.1. Search Strategy

We conducted a literature search in PubMed and ScienceDirect databases that identify any articles regarding bisphenol and its effects on the myometrium, decidua, placenta, and/or fetal membranes. We used the search terms: bisphenol AND (decidua OR placenta OR myometri* OR amnion OR chorion OR trophoblast) AND (human OR animal OR mouse OR mice OR sheep OR monkey OR cell OR “in vitro”) in the PubMed database. We used the search terms: bisphenol AND (decidua OR placenta OR myometrium OR amnion OR chorion OR trophoblast) in the ScienceDirect database. The search was performed last August 2021.

### 4.2. Inclusion Criteria

Since this is an integrative approach towards possible mechanisms of bisphenols contributing towards preterm birth, we included studies that would utilize bisphenol exposure on human or animal placental samples/animal models/tissue explants/in vitro cell cultures. We considered accessible, full-text articles that have been published in English or translated to English from 1999 to 2021.

### 4.3. Exclusion Criteria

We excluded articles that were individually assessed to be not related to mechanisms of preterm birth or parturition, as well as duplicate articles and review articles.

### 4.4. Article Strength Determination

We followed evidence determination similar to Peretz et al., 2014 [123]. Evidence was considered strong if multiple studies across different species indicate similar effect or outcome in a specific reproductive tissue, despite not 100% concordance to allow for species and strain differences. Evidence was considered limited when some studies, but not predominantly, indicate similar effect or outcome, or when discordant data is available across all studies. Lastly, evidence was considered inconclusive if only limited studies were performed in only one species or via in vitro studies alone.

## Figures and Tables

**Table 1:** Summary of studies delineating effects of BPA in the placenta.

Summary of Effects		References	Models Used	Strength	Notes
**Proliferation**	Increased—10^−5^–10 μM No effect—0.1–50 μM; Decreased—10^−4^–1 μM; ≥100 μM	Benachour 2007 Spagnoletti 2015 Morice 2011 Morck 2010 Basak 2018 Greca 2019 Greca 2020 Profita 2021	Embryonic 293 cells HTR-8/SVneo cells JEG-3, BeWo cells BeWo cells HTR-8/SVneo cells BeWo cells BeWo cells HTR-8/SVneo cells	STRONG	Different cell lines vary in the concentrations wherein increased proliferation occurs, but higher concentrations ≥ 100 μM are consistently cytotoxic Cell number of BPA-treated cells was significantly decreased when treated with G15, a GPr30 inhibitor
**Trophoblast motility**	Decreased migration—10^−5^–50 μM Decreased attachment—10–100 μM Decreased integrin, vimentin—0.1–10 μM Decreased E-cadherin—10^−3^–100 μM	Spagnoletti 2015 Lan 2017 Wang 2015 Gao 2019 Wei 2020	HTR-8/SVneo cells HTR-8/SVneo cells BeWo cells JEG-3 cells HTR-8/SVneo cells	STRONG	In HTR/SV8neo cells, membrane ER GPR30 antagonist, G15, partially restored HTR-8/SVneo trophoblast migration
**Placenta morphology**	Decreased labyrinthine zones, thinner spongiotrophoblasts—200 μg–50 mg/kg/day Decreased ratio of spongiotrophoblast zone to giant cell area—200 μg/kg Increased total vessel area—50 mg/kg/day	Tachibana 2007 Tait 2015 Lan 2017 Chu 2018 Mao 2020 Gwon 2020	Mouse placenta Mouse placenta HTR-8/SVneo cells JEG-3 cells Mouse placenta Mouse placenta	STRONG	
**Trophoblast morphology**	Increased syncytin and cell fusion—0.05 μM Decreased villous outgrowth—0.1 to 50 μM Increased villous outgrowth—10 μM Reduced syncytialization—0.5 mg/kg/day, 1 uM	Tait 2015 Narciso 2019 Gao 2019	Mouse placenta BeWo cells JEG-3 cells	STRONG	Increased villous outgrowth observed for trophoblast spheroid cell models; decreased villous outgrowth in 2D trophoblast cell cultures
**CRH production**	Increased CRH expression—200 mg/kg, 25–50 μM	Huang 2012 Tan 2013	JEG-3 cells Mouse placenta	STRONG	At 1–50 μM, increasing p-PKA, p-PKCalpha, which may mediate CRH expression
**Steroidogenesis**	Decreased CYP11A1, CYP19—10^−3^–100 μM Decreased progesterone, estradiol—10^−3^–1 μM Decreased aromatase—5–1000 μM Increased CYP1A1—10–50 μM No effect on serum progesterone in sheep—0.5 mg/kg/day	Benachour 2007 Nativelle-Serpentini 2003 Huang and Leung 2009 Chu 2018 Xu 2019	Embryonic 293 cells JEG-3 cells JEG-3 cells JEG-3 cells JEG-3 cells	LIMITED	In JEG-3 cells, no difference with CYP19 production
**MMPs**	Decreased MMP2, MMP9—10^−3^–100 μM Increased MMP9—0.1–10 μM	Wang 2015 Wei 2020	BeWo cells HTR-8/SVneo cells	LIMITED	
**TIMP**	Increased TIMP1, TIMP2—10^−3^–100 μM Decreased TIMP3—10–50 μM	Lan 2017 Wang 2015 Wei 2020	HTR-8/SVneo cells BeWo cells HTR-8/SVneo cells	LIMITED	
**miR-146a**	Increased—25 ng/μL	Avissar-Whiting 2010 de Felice 2015	SV40 cells Human placenta	LIMITED	miR-146a was the only miRNA validated by qRT-PCR as significantly upregulated in both 3A and HTR-8 cells with BPA treatment at 25 ng/μL
**Other hormone production**	Decreased serotonin—200 μg/kg Increased dopamine—200 μg/kg Decreased B-hCG—10^3^–10^8^ μM Increased testosterone concentrations—20–200 mg/kg/day	Morck 2010 Tan 2013 Mao 2020	BeWo cells Mouse placenta Mouse placenta	LIMITED	Not necessarily concluding towards preterm birth
**Apoptosis-related**	Increased apoptosis—0.01–0.1 μM Increase caspase-3 production—10^−6^ μM Decreased caspase-3 production—9 μM	Morice 2011 Narciso 2019	JEG-3, BeWo cells BeWo cells	INCONCLUSIVE	
**Oxidative stress**	Increased ROS production—50–500 μM Increased HSP70, Bcl2—0.9 to 9.0 μM Decreased GSH—0.9 to 9.0 μM Increased Nrf—9.0 μM	Ponniah 2015 Perez-Albaladejo 2017	BeWo cells JEG-3 cells	INCONCLUSIVE	Decrease in GSH + increased ROS production increases oxidative damage, while Nrf, HSP70, and Bcl2 production may be counterregulatory measures
**Inflammation**	Decreased TNF-alpha—1.4 × 10^−6^ to 0.04 μM Increased IL-6—1–10 μM Increased IL-1B—0.001–10 μM	Benachour and Aziz 2009 Arita 2019	Human primary cytotrophoblasts Placental explants	INCONCLUSIVE	
**PR receptor**	Decrease in PR expression—2 μg/kg/day	Imanishi 2003	Mouse placenta	INCONCLUSIVE	Decrease in PR expression was embryo sex-selective
**ER receptor**	BPA binds to ERRg about 100 times greater than to ERa and Erb	Takeda 2009	Human placenta	INCONCLUSIVE	May provide alternative hypothesis for a pathway for other BPA actions other than classical steroid receptors
**ERK1/2**	Increased p-ERK—10^−3^–1 μM	Chu 2018 Lan 2017	JEG-3 cells HTR-8/SVneo cells	INCONCLUSIVE	treatment with the extracellular signal-regulated kinase (ERK1/2) inhibitor U0126 or the PI3K inhibitor LY294002 for 24 h abolished the BPA-induced phosphorylation of ERK and Akt
**Akt**	Increased p-Akt—0.01 μM	Lan 2017 Greca 2020	HTR-8/SVneo cells BeWo cells	INCONCLUSIVE	treatment with the extracellular signal-regulated kinase (ERK1/2) inhibitor U0126 or the PI3K inhibitor LY294002 for 24 h abolished the BPA-induced phosphorylation of ERK and Akt
**PAG1 and PSPB**	Decreased serum concentration—0.5 mg/kg/day	Gingrich 2018	Sheep placenta	INCONCLUSIVE	
**Exosomes**	Increased placental exosome expression of HMGB1, caspase 4, MAPK14 expression—20 μM	Sheller-Miller 2020	Human placental exosomes	INCONCLUSIVE	

**Table 2:** Summary of studies delineating effects of BPA in the decidua.

Summary of Effects		References	Model Used	Strength	Notes
**Inflammation**	Increased LIF, IL-10—10 μM Decreased PAI-1, TNFa—10 μM Decreased CXCL8, IL-6, CCL11—1–10 μM Increased CXCL6—1–10 μM Increased IL-6—10^−6^–0.001 μmol Increased I-kB, nF-kB, IL-1B—10^−6^–0.001 μmol	Li 2017 Cho 2018 Fan 2020 Xiong 2020	Human decidual stromal cells Human endometrial (EM) cells Human EM cells, JEG-3 cells Human EM cells	STRONG	Effects on IL-6 may be discordant due to two different laboratory setups; additionally, total concentration of BPA was not noted in Cho 2018, precluding us from identifying at what exact concentration is the IL-6, I-kB, nF-kB, and IL-1B increase noted.
**ER expression**	Decreased—50–100 μM, 0.5 mg/kg/day–20 mg/kg/day Increased ERa—10^−6^–1 μM Increased ERB—0.001 μM	Varayoud 2008 Aghajanova and Giudice 2011 Mannelli 2015 Cho 2018	Human uterine stromal cells Human EM cells Placental explant Human EM cells	STRONG	Total concentration of BPA was not noted in Cho 2018, precluding us from identifying at what exact concentration is the ERa increase noted. SMRT corepressor decreases ER and PR receptivity as per Varayoud 2008
**PR expression**	Increased—50 μg/kg/day, 1 μM	Aldad 2011	Human EM cells, Ishikawa cells	LIMITED	SMRT corepressor decreases ER and PR receptivity as per Varayoud 2008 No effect as per Aghajanova and Giudice 2011
**Decidual proliferation**	Decreased—50–100 μM, 0.5 mg/kg/day to 20 mg/kg/day Increased—0.01 μM	Varayoud 2008 Aghajanova and Giudice 2011	Human uterine stromal cells Human EM cells	LIMITED	No effect on viability as per Mannelli et al.; may be due to differences in setups
**Steroidogenesis**	Decreased P450scc—50–100 μM Decreased 17BHSD1—50–100 μM Increased SMRT—0.5 mg/kg/day and 20 mg/kg/day	Aghajanova and Giudice 2011 Varayoud 2008	Human EM cells Human uterine stromal cells	LIMITED	
**IGFBP-1**	Increased—10^−8^ to 10^−11^ μM, 50 μM	Aghajanova and Giudice 2011 Forte 2015 Fan 2020	Human EM cells Human EM cells Human EM cells, Ishikawa cells	LIMITED	No effect on IGFBP-1 secretion as per Mannelli et al.; may be due to differences in setups
**PRL**	Increased—10^−8^–10^−11^ μM, 0.1–10 uM Decreased—0.1–88 μM	Forte 2015 Mannelli 2015 Olson 2017 Fan 2020 Xiong 2020	Human EM cells Placental explants HuF cells Human EM cells, Ishikawa cells Human EM cells	LIMITED	Same endometrial cells, but different effects, perhaps due to different laboratory setups At lower concentrations, PRL appears to be increased, while for higher concentrations, PRL appears to be decreased
**ENaC**	Decreased—0.01–1 μg/mL	Yuan 2018	Mouse EM cells, Ishikawa cells	INCONCLUSIVE	
**SGK1**	Decreased—100 μg/kg	Yuan 2018	Mouse EM cells, Ishikawa cells	INCONCLUSIVE	
**Cell cycle**	Lower G0/G1 cycles, Higher G2/M cycles—10^−11^–10^−8^ μM Decreased CCND2—44 μM	Forte 2015 Olson 2017	Human EM cells HuF cells	INCONCLUSIVE	
**Hoxa10/11**	Decreased—0.5–20 mg/kg/day	Varayoud 2008	Human uterine stromal cells	INCONCLUSIVE	
**Egr1**	Increased—20–500 mg/kg	Kim 2017	Mouse uterus	INCONCLUSIVE	ERK1/2 and AKT were rapidly activated by BPA, BPA-induced Egr1 expression is mediated by ERK1/2, but not AKT phosphorylation
**p-ERK**	Increased—10^−6^–0.001 μmol	Cho 2018	Human EM cells	INCONCLUSIVE	Both appear to increase similarly, but ERK may be more involved in the signaling process than Akt (Kim 2017)
**p-Akt**	Increased—10^−6^–0.001 μmol	Cho 2018	Human EM cells	INCONCLUSIVE	
**p-JNK**	Increased—10^−6^–0.001 μmol	Cho 2018	Human EM cells	INCONCLUSIVE	
**Histone methylation**	Decreased MLL1—0.1–10 μM Increased EZH2—0.1–10 μM Increased H3K4me3—0.1–10 μM Increased H3K27me3—10^−5^–10 μM	Xiong 2020	Human EM cells	INCONCLUSIVE	
**Oxidative stress**	Decreased SOD, catalase, HO—10^−6^–0.001 μmol	Cho 2018	Human EM cells	INCONCLUSIVE	Total concentration of BPA was not noted in this experiment; only picomolar values were given

**Table 3:** Summary of studies delineating effects of bisphenols in the myometrium.

Summary of Effects		References	Model Used	Strength	Notes
**Uterine weight and thickness**	Increased—2–30 mg/kg/day, 5 mL/kg Increased thickness—50 mg/kg/day Increased endometrial area—3.5 mg/kg Increased luminal epithelium height—2 mg/day	Dodge 1996 Ashby and Tinwell 1998 Papaconstantinou 2000 Okuda 2010	Mouse uterus Mouse uterus Mouse uterus Human myometrial cells	STRONG	No effect on uterus weight and histological parameters as per Okuda; differences may be due to difference in lab setups EM thickness increase in ewe lambs; luminal height in mice
**Uterine contractility**	Decreased—10^−6^–10^−4^ μM Decreased amplitude—1–10 μM	An 2013 Gupta and Deshpande 2018 Salleh 2015	Rat uterus Rat uterus Rat uterus	STRONG	
**VACht fibers**	Increased—0.05 and 0.5 mg/kg/day	Liliana 2019	Pig uterus	LIMITED	
**DBH fibers**	Increased—0.05 and 0.5 mg/kg/day	Liliana 2019	Pig uterus	LIMITED	
**Implantation rates**	Decreased—100 mg/kg/day Decreased—implantation sites—6.75 and 10.125 mg/kg/day Delayed embryo reception—40 mg/kg/day	Xiao 2011 Berger 2010	Mouse myometrial cells Mouse myometrial cells	LIMITED	
**hsp27**	Increased—100 mg/kg/day	Papaconstantinou 2001	Mouse uterus	INCONCLUSIVE	
**grp94**	Increased—100 mg/kg/day	Papaconstantinou 2002	Mouse uterus	INCONCLUSIVE	
**oxytocin and oxytocin receptor**	Increased oxytocin—500 mg/kg/day Increased oxytocin receptor—100 mg/kg/day	An 2013	Rat uterus	INCONCLUSIVE	
**PR expression**	Decreased—100 mg/kg/day Decreased—10.125 mg/kg/day	Xiao 2011 Berger 2010	Mouse myometrial cells Mouse myometrial cells	INCONCLUSIVE	
**ER expression**	Decreased—10.125 mg/kg/day	Berger 2010	Mouse myometrial cells	INCONCLUSIVE	
**Gene expression profile**	Increased genes for PI3K-Akt signaling, metabolic pathways, cancer pathways, actin regulation, ECM interaction, focal adhesion—0.01 μM	Kang 2014	Human myometrial cells	INCONCLUSIVE	

## Data Availability

Not applicable.
